# “Our girls need to see a path to the future” --perspectives on sexual and reproductive health information among adolescent girls, guardians, and initiation counselors in Mulanje district, Malawi

**DOI:** 10.1186/s12978-018-0661-x

**Published:** 2019-01-25

**Authors:** Kristin Nash, Gabrielle O’Malley, Elizabeth Geoffroy, Ellen Schell, Alice Bvumbwe, Donna M. Denno

**Affiliations:** 1 Department of Health Services, School of Public Health, University of Washington, Seattle, WA USA; 2 Department of Global Health, School of Public Health, University of Washington, Seattle, WA 98104 USA; 3 Global AIDS Interfaith Alliance (GAIA), 2171 Francisco Blvd. E., Suite I, San Rafael, CA 94901 USA; 4 Global AIDS Interfaith Alliance (GAIA), Blantyre, Malawi; 5 Department of Pediatrics, School of Medicine, University of Washington, 6200 NE 74th Street, Bldg. 29, Box 354920, Suite 110, Seattle, WA 98115 USA

**Keywords:** Adolescent girls, Contraception, Cultural inconsistencies, Early pregnancy, Information, education, and communication (IEC), Initiation ceremonies, Malawi, Mulanje District, Multiple stakeholders, Role models, Sexual and reproductive health (SRH), Sub-Saharan Africa, Transactional sex, Unintended adolescent pregnancy, Youth friendly SRH services

## Abstract

**Background:**

Malawi has one of the highest adolescent pregnancy rates worldwide; at 141 births/1000 girls it is 3-fold higher than the global average. Adolescent pregnancy contributes to poor maternal and neonatal outcomes, school dropout, and poverty. In preparation for an information, education, and communication (IEC) intervention to reduce unintended pregnancy among adolescent girls, formative research was conducted to understand how and what sexual and reproductive health (SRH) information is shared with girls in southern, rural Malawi, and perceptions of such information among key informants.

**Methods:**

Forty semi-structured interviews were conducted with three participant groups: adolescent girls (*n* = 18), mothers/female guardians (M/FGs) of adolescent girls (*n* = 12), and leaders of initiation rites (*n* = 10). Interviews were conducted in 15 villages. Data were analyzed and coded using Dedoose 7.5.

**Results:**

Participants widely acknowledged both the health risks and the general social unacceptability of early childbearing, yet adolescent pregnancy is common in the region. Respondents also acknowledged the importance of female school completion and the norm that pregnancy usually marks the end of a girl’s education. Unprotected transactional sex was reported to be common and driven by poverty. Initiation rites were described as prevalent and often encourage girls to practice sex at puberty. Contraceptives, and even condoms, were reportedly discouraged for adolescents due to concerns about inappropriateness for nulliparous and young girls and misconceptions about side effects. Adolescent respondents also noted barriers to accessing condoms and contraceptives. M/FGs were described as gatekeepers to SRH information and services, and many parents reported delaying SRH discussions until after sexual debut due to concerns about encouraging sexual activity. Adolescent and M/FG participants expressed a desire for role models or “outside experts” to provide SRH education and to promote an alternate vision to adolescent motherhood.

**Conclusion:**

To improve SRH outcomes for adolescent girls, it is critical to engage key stakeholders and create an enabling environment so that girls can effectively act on the IEC they receive. Initiation counselors remain entrenched information sources; efforts to provide them with training on accurate SRH messaging could leverage an existing channel. Engaging parents, especially mothers, is crucial to encourage earlier SRH education and to gain their acceptance of adolescent access to SRH services. Also important is mobilizing the broader community of influencers in support of girls’ SRH and vision for a healthier future. Sensitization messages focusing on the health, educational and economic benefits of preventing early pregnancy may overcome misconceptions about and barriers to contraceptive use. Finally, fostering girls’ aspirations for school completion and jobs and other income generating opportunities via role models can encourage an alternative to adolescent motherhood. Ultimately, poverty and gender inequity reduction is critical for long-lasting impact on the SRH of adolescent girls in the region.

## Plain English summary

Adolescent pregnancy rates are high across sub-Saharan Africa, and more so in Malawi, one of the poorest countries in the world and an HIV hotspot. Childbearing in adolescence is associated with poorer health outcomes, lower education attainment, and lower socioeconomic status for the mother and her offspring. The current study was undertaken to inform a program to reduce unintended adolescent pregnancy in a rural, low-literacy African setting. We sought to better understand the content and sources of sexual and reproductive health information for adolescent girls, as well as to identify preferred information channels through in-depth interviews with girls, mothers/female guardians and leaders of initiation ceremonies that take place at puberty. Participants strongly believed that childbearing before age 18 is undesirable and staying in school is important. However, they also reported that initiation ceremonies are widespread and often promote intercourse, early transactional sex is common and driven by poverty, and use of contraceptives and even condoms among young and nulliparous girls is discouraged. This reveals a cultural inconsistency and also an opportunity to leverage facilitators to reduce early pregnancy and its adverse health and economic consequences. The results of this formative study suggest ways forward, including the importance of engaging multiple community stakeholders in a shared effort.

## Background

Globally, approximately 16 million girls ages 15–19 and two million under-15 years become pregnant each year, the vast majority among the poorest in low- and middle-income countries (LMICs) [1]. It is estimated that more than 12 million adolescent girls (defined as 10–19 years of age) give birth annually [2]. Pregnancy and childbirth among adolescents are associated with higher risks of poor maternal and neonatal health outcomes compared to women aged 20–24 [3]. High adolescent pregnancy rates create continued economic burden and perpetuate cycles of poverty. For example, in a study of five LMICs, children of adolescent mothers had 30–40% greater odds of failing to complete secondary school -- a major determinant of lower socioeconomic status – compared to older mothers [4].

High adolescent birth rates contribute to a fast-growing and proportionally young population in sub-Saharan Africa (SSA). In Malawi, more than a quarter of the population is aged 10–19, [5] and the adolescent birth rate is approximately 31% overall -- [6] the seventh highest globally – and 44% among the poorest quintile. [7] Notably, one-third of all new HIV infections are among 15–24 year olds, with 70% among girls and young women [8]. At the current growth rate, Malawi’s population could more than double by 2050, posing immense challenges to development efforts [9]. Specifically, high rates of pregnancy and HIV infection among a growing adolescent population will impact Malawi’s ability to capitalize on the demographic dividend – a window of opportunity to achieve economic growth due to declining mortality and fertility rates that increase the working-age population in relation to number of young dependents [10].

In addition, early pregnancy is among the primary reasons for school dropout among girls [11]. According to Malawi’s Ministry of Education, Science and Technology (MoEST) policy, girls face a compulsory 12-month leave-of-absence when they become pregnant [12]. This contributes to low school completion rates among girls in Malawi, with 9.8% of females aged 20–24 having completed secondary school. Educational attainment is lowest among those in the lowest wealth quintile and rural dwellers, where women have a median of 1.6 and 2.7 years of schooling, respectively [11]. In 1994, Malawi introduced free primary school and enrollment increased by more than 50% the next year, overburdening the system and impacting the quality of education [13]. The MoEST recently announced that, effective January 2019, school fees for conventional and community day secondary schools (not for boarding schools) will be abolished.

Several factors contribute to high adolescent fertility rates in Malawi and similar settings, including lack of SRH knowledge, limited access to/use of contraceptives, condoms, and SRH services, gender inequality and cultural practices such as child marriage and initiation ceremonies. Notably, in Malawi, 98% of 15–19 year old girls are aware of modern methods of contraception, yet only about one-third of sexually active unmarried girls ages 15–19 use them [14]. Reasons given for lack of uptake include religious/cultural beliefs, negative attitude of clinic staff, inability to negotiate use with partners, and misconceptions including that contraceptives cause cancer or infertility. Limited access to SRH services and commodities is an impediment that the Government of Malawi has sought to address. In 2007 the first National Youth Friendly Health Services Strategy was launched and aimed at delivering services that are relevant, accessible, attractive, affordable, appropriate and acceptable to young people [15]. However, a 2014 evaluation found that only about 32% of young people had heard of YFHS and 13% had ever used these services [16].

Malawi ranks in the bottom quintile of countries on the Gender Inequality Index, which is a composite measure of reproductive health, female empowerment and economic status. Gender inequality contributes to low decision-making power among girls, including the ability to negotiate safe sex. For example, 38% of Malawian girls ages 12–19 reported that their first sexual experience was forced or coerced, according to a household survey [17]. And while the legal age of marriage was set at 18 in 2017, the law is inconsistently enforced. Child marriage -- defined by the United Nations as marriage under age 18 -- is common, especially in rural southern Malawi where about half of females marry before age 18 [11].

Initiation ceremonies in Malawi play an important role in teaching about traditional practices and sexual expectations. Such ceremonies, especially prevalent in the rural south where about 57% of adolescent girls participate, [18] are connected to menarche, marking pubertal transition. The literature is scant on the content covered during these initiation rites, but traditional song and dance is taught and girls are educated on topics such as personal hygiene and respect for elders and caring for family, as well as how to please sexual partners. “Cleansing the dust,” which is to practice sex soon after the initiation to symbolize the transition to adulthood, is also often encouraged [19]. More recently, some churches have created their own ceremonies apart from the traditional ones that purportedly discourage sex before marriage.

Comprehensive sexuality education (CSE) delivered through a multi-year, rights-based, and gender-focused approach is critical for improving SRH knowledge and life skills [20]. The Government of Malawi committed to implementing quality CSE when it signed UNESCO’s Ministerial Commitment for adolescents and young people in Eastern and Southern Africa. However, in Malawi, CSE is delivered at the secondary school level, usually in year three, when the majority of girls already have dropped out of school and roughly half have experienced sexual debut [11]. Furthermore, CSE implementation is inconsistent and there are content gaps, especially related to pregnancy prevention. Other barriers include lack of parent and community support due to lack of awareness regarding its value and parental demands that their children not be exposed to “obscenities” [12].

Global AIDS Interfaith Alliance (GAIA) is a non-governmental organization that has been supporting HIV prevention and treatment, as well as basic health care in Mulanje District, located in rural Southern Malawi, via mobile clinics, counseling, and information, education and communication (IEC) activities since 2000. GAIA has focused in Mulanje due to the district’s high HIV prevalence coupled with poor access to health care. The low social status of girls and persistent high rates of unintended pregnancy and their impact on health and socioeconomic outcomes has prompted GAIA to consider a SRH IEC and girls empowerment intervention. The effort will be framed within a CSE context, emphasizing gender, agency and rights in order to increase empowerment as well as reduce rates of unintended early pregnancy. The current study was designed to inform development of programming by GAIA and other groups in rural sub-Saharan African settings. Recognizing that adolescent SRH-related behaviors are influenced by a range of social relationships and institutions, we sought to understand the existing content of SRH information delivered to girls in the setting, as well as their preferred sources and ways of receiving such information.

## Methods

### Data collection

We conducted 40 key informant interviews in 15 villages in Mulanje. A GAIA coordinator assisted the research team in choosing the villages, based on geographic and religious diversity, as well as accessibility. We recruited three types of interview participants:

- adolescent girls, ages 10–18 (*N* = 18)
- mothers/female guardians (M/FGs) of 10–18 year-old girls living in the household (*N* = 12)
- traditional and/or religious initiation counselors for adolescent girls (*N* = 10)

We recruited adolescents up to age 18, as this is the legal age of marriage. In addition, while we set out to include younger adolescents down to age 10, a higher proportion of girls in this age group declined to participate. As a result, the older adolescent sub-group had twice (*n* = 12) as many participants as the early adolescent (ages 10–14) sub-group (*n* = 6). We included twelve M/FG participants; another five declined due to scheduling conflicts. Of the 10 initiation counselors interviewed, four identified as religious counselors, one as a traditional counselor, and five as both religious and traditional. They all reported beginning as traditional counselors. No counselors or older adolescent girls refused to participate. The interviews in each participant category were conducted until informational saturation was reached [21].

Three semi-structured interview topic guides were adapted from tools developed and validated by the World Health Organization, [22] and translated into Chichewa, the predominant local language. Areas explored included: pubertal changes and menses; sexual expectations and behavior; early pregnancy and childbirth; contraception and condom use; as well as sources of SRH information including initiation ceremonies. We also asked about the value of education for girls. The interview guides included open-ended questions in each of these areas, making no assumptions about knowledge levels, attitudes or beliefs. For each area, we commenced with asking girls what they knew about that topic. For example, “I’m interested in what you know about growing up and bodily changes,” followed by questions regarding from whom and where they learned the information (e.g., parents, school, media, peers), and about their preferred information sources.

Village chiefs provided lists of households with adolescent girls, generally 15–20 households per village. Going door-to-door, we recruited girls and M/FGs based on who was home at the time of our visit, aiming for geographic and demographic diversity. Village chiefs and GAIA coordinators provided lists of traditional and religious counselors who were visited at home in similar fashion.

The principal investigator (KN) along with a team of eight local interviewers -- mostly females, ranging in age from 22 to 30 -- conducted face-to-face interviews in Chichewa in April 2016. They were trained to establish rapport, make the key informants feel as comfortable as possible, and remind them that they could ask questions or opt out at any time. Individual interviews lasted approximately 45 to 60 min, with some going longer to accommodate participant responses.

All interviews were audio-recorded, transcribed, translated into English, and coded using Dedoose 7.5. Clusters of coded data were reviewed alone and in relation to the overall data set. An inductive approach was used to identify overarching themes and sub-themes.

Verbal informed consent/assent was obtained from each participant. The study protocol was approved by the University of Washington (Human Subjects Application #50229) and the Malawi National Health Sciences Research Committee (Protocol/Approval #16/3/1546).

## Results

In addition to qualitative data, demographic information was collected for each of the three participant groups (Table 1). Notably, four girls reported a previous pregnancy, all of which were unintended. Of the five girls who dropped out of school, four did so due to pregnancy and one due to lack of funds. The girls had a higher average level of education, having completed 7.2 years, versus 3.4 and 1.8 for M/FGs and initiation counselors, respectively. Ninety-percent of initiation counselors reported having no formal training to conduct initiation ceremonies. Qualitative analysis identified seven major thematic areas, which are listed and described below, with supplementary participant quotes presented in Table 2.

**Table 1:** Summary Demographic Data

Adolescent Girls (*N* = 18)		
	N (%) or Mean (SD, range)	
Age		
Early adolescent (age 10–14)	6 (33.3)	
Late adolescent (age 15–18)	12 (66.7)	
Average age	15.6 (1.8, 13–18)	
Average years of education	7.2 (1.4, 5–10)	
Level of education^1^		
Some primary	10 (55.5)	
Completed primary	6 (33.3)	
Some secondary	2 (11.1)	
School drop-out	5 (27.7)	
Reasons for school drop-out		
Pregnancy	4 (80)	
Lack of finances	1 (20)	
Ever been married^2^	3 (16.6)	
Average age at marriage	16.3 (1.5, 15–18)	
Ever been pregnant^3^	4 (22.2)	
Unintended pregnancy	4 (100)	
Average age at first pregnancy	15.3 (1.7, 13–17)	
*1.Educational attainment in Malawi (girls age 15–19, as per DHS age cohort, DHS 2015–16):* *Some primary: 65.4%* *Completed primary: 5.7%* *Some secondary: 23%* *Completed secondary: 2.6%* *2. 47% of women (25–49 years) in Malawi were married by age 18 (Malawi DHS 2015–16).* *3. 29% of 15–19 year-old girls have begun childbearing in Malawi (DHS 2015–16), an increase compared to 25.6% from 2010 DHS data.*		
Mothers/Female Guardians of Adolescent Girls (N = 12)		
		Median (SD, range)
Average age	50.8	46 (15.5, 32–80)
Average years of education	3.4	3 (3.4, 0–8)
Average age at marriage	19.3	18 (3.4,15–26)
Average age at first pregnancy	19.4	18 (3.7,16–28)
Average number of pregnancies	7	6.5 (3.9, 2–17)
Average number of living children	5.1	5 (1.8, 2–8)
Initiation Counselors (N = 10)		
	N (%)	Median (SD, range)
Average age	59	60 (9.2, 45–70)
Average years of education	1.8	1.5 (1.69, 0–5)
Religious counselor	4 (40)	
Traditional counselor	1 (10)	
Traditional and religious counselor	5 (50)	
Personal religious affiliation		
Christian	6 (60)	
Muslim	3 (30)	
None	1 (10)	
Received formal training as an initiation counselor	1 (10)

**Table 2:** Additional Participant Quotes by Theme

*Broad awareness that early pregnancy can be harmful to adolescent girls*	*We tell them that if they happen to deliver using the normal way, the passage area may tear and they may begin to urinate uncontrollably. Due to such a problem, one may never get married. That is what we tell them. Initiation Counselor; ZDB01* *If they become pregnant whilst still young, when they go to give birth, a knife is used to cut their belly and remove the baby inside them because it is impossible to deliver normally. Initiation Counselor; ZDB01* *It is not good to give birth before 18; there are serious complications that happen when you give birth before 18, I tell them they can die, they can miscarry. M/FG; YAA02* *If a girl has sex with a man she will get pregnant or HIV and other sexually transmitted diseases. She may even become barren or die from complications of giving birth if the mother is under 18. M/FG; YAA02* *They say when you get pregnant before 18 you go through a lot of complications, because the body parts are not yet strong enough or matured to give birth. 14-year-old girl; XKB02* *Girls who give birth while young [before age 18] can give birth through Cesarean and can have a pre-mature birth. 18-year-old girl; XGA01*
*Gender norms and poverty encourage early sexual debut*	*At [age] 8 they have already started sleeping with men to get financial and material support to get their needs met.* M/FG YAA03 *Sometimes girls do have many relationships because they want money to fulfill their heart’s desires.* MF/G; YCA02 *Most men in this village do business and they take that as an advantage that they have money and can propose any young girl and the girls don’t say no because they are attracted to the money they are given.* MF/G; YCC01 *Most of them expect us to have sex with them whenever they feel like having, and when they get them [girls] pregnant they start telling them to abort.* 18-year-old girl; XKA04 *They impregnate a girl and he starts to reject the pregnancy and the boy starts insulting the girl. A girl cannot deny [sex] because she is afraid that the boy will not be giving money to her.* 15-year-old-girl; XBA01 *Many boys want to be in a relationship with you because you are still at school, they promise so many things that they don’t fulfill. What they want is to have sex with you and get you pregnant, at the end they deny the pregnancy.* 15-year-old girl; XBC01
*Female education is valued, but threatened by pregnancy*	*[I dropped out of school because] my parents were not able to provide my needs, personal needs. I was admiring my friends so I decided to have a relationship to give me money to fulfill my heart’s desires and later found myself pregnant.* M/FG; YCB03 *I have learned that you are not supposed to start having sex while still in school. You are supposed to start after finishing secondary school. [If you have sex] you can get pregnant and drop out of school; you can get HIV/AIDS.* 14-year-old-girl; XKA01 *The boys impregnate a girl and then leave her. She has to leave school. Girls may want to return [to school] after, but that is very difficult. 14-year-old girl; XKB02* *My parents could not afford to raise money to pay school fees for all of us in the family, I dropped out and got pregnant. M/FG; YCA01*
*Desire for outside experts as teachers and role models*	*Yes, there will be a change [fewer pregnancies among girls under 18] because they will realize the importance of a message coming from people who live far away.* M/FG; YDA02 *When they [GAIA women, counselors,* etc.*] are educating us, many people especially girls, gather so this helps us to exchange information and we learn more.* 16-year-old girl; XBS01 *When the social counselors come into the picture, they [girls] tend to begin valuing such advice as true and helpful.* M/FG; YDA02 *Girls admire the life of the one telling them [social counselor/ role model], therefore they listen and do according to what they have been advised, thinking that perhaps their lives will become more like the life of the counselor.* M/FG; YDA02 *There must be regular meetings about these issues in the villages and role models must come as a way of convincing the girls.* M/FG; YFB01 *If mentorship programs can be added, girls can get encouraged to learn from them, concentrate in school and become like them.* M/FG; YCC01
*A range of impediments to contraceptive use exist*	*I tell them contraception ways can look attractive but they are dangerous to girls who are not yet married. They have their own side effects that can make them never to bear children in the future.* M/FG; YKB03 *We don’t tell them the use of condoms [at initiation ceremonies], condoms are not trustworthy… Condom doesn’t make any sense to me.* Initiation counselor; ZFA02 *It’s not necessary for them between 10 to 18 years of age [to learn about contraception]. They are supposed to wait until their time comes… In initiation camps, no one can tell them the use of condoms or contraception.* Initiation counselor; ZFC01 *Girls should learn, but they should not be given contraceptives, because using contraception will make them to have problems in future, like damage to the uterus.* 18-year-old-girl; XBA02 13-year-old girl; XBB01
*SRH education starts too late*	*If they hear more about this [SRH] at a tender age, it disturbs their school plans. It really gives problems to those under 18 of age because the information reaches their shallowest point of their mind due to immaturity.* MF/G; YFA01 *Actually, it [providing SRH information] is only when we start observing their movements. For example, a child could just leave the house… and returns very late. She sometimes answers in a challenging manner when questioned and sometimes gives rude remarks [indicating she has begun to have sex].* M/FG; YDC02 *At [age] 8 they have already started sleeping with men, so according to me age does not matter but maybe if parents start advising them while they are still young even before they reach 8.* M/FG; YCC01 *Most parents start counseling their children at 15, missing it at 10, 11, 12, 13, 14. To start counseling them at 15, it’s too late, and within 2–3 years you will find out that she is pregnant. So maybe the counseling can start as early as 8 years old.* M/FG; YCB03 *I tell them that when they have come of age, starting to experience their menstruation period, it means they are now in a danger zone, but actually I don’t sit them down to tell them the actual details because it’s not appropriate at this stage.* MF/G; YFA01
*Initiation practices that encourage sexual activity*	*If you go for initiation, they told you to sleep with a man after you have been initiated. So if you didn’t have any idea before, you want to try sex because you have been initiated.* 18-year-old-girl; XBA02 *At traditional initiation camps, they [girls] are told to bend and dance in a sexual manner. When that girl comes out of the initiation camp she will begin having sex because that is what she has been taught. They are taught how men perform in bed.* Initiation counselor; ZDB01 *We tell them to remove dust after finishing an initiation to cleanse themselves. Removing dust is our traditional way of doing things where by on the day of releasing the girls from the camps, men are arranged to have sex with them in that way they have been cleansed.* Initiation counselor; ZCC02 *They are taught that after initiation they should go and have sex for the first time, which is called sexual cleansing.* Initiation counselor; ZZA01 M/FG; YWA02 *I think girls must not be sent to camps because when they come back they are completely changed, they go astray. But if a parent can take part in the counseling process it can be good.* M/FG; YAA02

### Broad awareness that early pregnancy can be harmful

Even the youngest girls, along with caregivers and counselors, were familiar with the notion that childbearing while young can result in harmful health outcomes for mother and baby. Depth of knowledge differed, but many could name specific risks, including fistula, C-section, preterm birth and death.

*“It’s not good for girls to fall pregnant before 18, that’s what I can say. I tell them that if they get pregnant before they are old enough, they might experience complications in giving birth... I tell her she can get fistula, I even tell her she can die with the baby together during the delivering process.”* M/FG; YCC01.

Although participants were universally against childbearing before age 18, nearly all reported that childbearing before age 18 is common in their village.

### Gender norms and poverty encourage early sexual debut

All three participant groups said that girls under 18 commonly accept money and gifts in exchange for sex, which is typically unprotected, often with multiple cross-generational sexual partners, and that this exchange encourages early sexual debut, sometimes as young as 8 years old. Participants reported that poverty often drives transactional sex for coverage of basic needs like food, soap and clothing:

*“Most of the time it [girls having sex] is because of poverty. Let’s say you are lacking resources at home, like soap, food, a lot of things. You will do it.”* 18-year-old girl; XKC02.

However, aside from basic necessities, participants also described how gifts from sexual partners enable girls to obtain their “heart’s desires,” for example cell phones.

*“Sometimes a girl might take boyfriends just for nice things that she could not have, like to get hair done or even a phone.” 18-year-old girl; XBA02.*

Most girls in this study reported not being able to say “no” to males when it comes to sex, because they need money and/or they feel threatened by violence.

*The boy has spent a lot of money on you and it is not for you to deny when he wants to have sex; you can’t reject because he can beat you. 18-year-old girl; XBA02.*

Some girls related that boys want to impregnate them and then leave them, often denying that the baby is theirs altogether.

*“The boys just want to impregnate the girl and leave her and they expect girls to say yes every time they want to have sex.” 13-year-old girl; XBB01.*

### Female education is valued, but threatened by pregnancy

Despite their own low educational attainment, M/FGs and initiation counselors almost universally stated that a girl’s education is important for her own economic future and that of her family.

*“We tell them to work hard at school so that their future should be brighter… a girl should go to school and be well educated so that in the end we parents should be helped by our girls.”* M/FG; YAA03.

Girls reported that they are taught the value of staying in school, but that motherhood marks the end of a girl’s education. All four of the adolescent girl participants who had borne children reported dropping out of school due to the pregnancy. A few participants expressed that girls should be able to return to school after giving birth, but that this is extremely difficult and only possible when family members can care for the child and support the girl.

*“When a girl is in this situation [pregnant], after difficulties to look after her baby, she ends up asking her parents to allow her to go back to school.” M/FG; YAA03.*

Participants reported that the burden of adolescent childrearing falls primarily on the girl, and noted that when girls become pregnant they drop out of school, while the boy is able to remain in school.

*“[The girls] will drop out of school, while the boys continue with school. They [girls] will be the ones suffering with the child without proper financial help from the father who is also a child and this becomes a burden to us too as parents.”* M/FG; YCC01.

### Desire for outside experts as teachers and role models

While acknowledged among all participant groups that mothers/guardians are gatekeepers to their daughters’ access to SRH information and services, girls expressed two potentially conflicting preferences. On the one hand, girls reported that they value talking to M/FGs about SRH issues because mothers have their best interests in mind and girls can ask questions.

*“Mother is best because she is the one I am close with and open and because I see her daily makes it easy.”* 14-year-old girl; XKB01.

At the same time, girls and M/FGs reported that parents often lack credibility with their daughters, and information from outside experts would be better heeded due to the perceived increased knowledge and expertise of someone from “far away”:

*As we are staying together we become used to each other and they [girls] become rude and challenging and don’t listen to us [parents]. M/FG; YFA01.*

*There will be a change [fewer adolescent pregnancies] because girls will realize the importance of a message coming from people who live far away from their community. M/FG; YDA02.*

Importantly, outside experts and role models were suggested by girls and M/FGs not only for their perceived credibility, but also for their ability to help girls develop aspirations for the future. Many reported that in the village girls see motherhood as the only option and – while education is valued in the community overall – without an alternative, they often lose interest in completing school. Participants suggested that female role models coming to the village would give them a sense of future possibilities related to education and delayed childbearing.

*“Girls know school is right, but without opportunities [to earn a living] they lose hope... Role models must come as a way of convincing the girls… the girls admire the one telling them, therefore they listen and do accordingly… thinking that perhaps their lives will become more like the life of the counselor.” M/FG; YCA02.*

Though not all girls had received sexual education in school, girls reported that they would like/like to learn about SRH topics from teachers (especially female teachers) in school because the information is perceived to be reliable and they can ask questions.

*“At the initiation camp, we are not allowed to ask any questions. At school, you can learn about sexual intercourse and be able to ask the teacher where you don’t understand and where you want to get more information.” 14-year-old girl; XKA01.*

### A range of impediments to contraceptive use exist

The majority of adult participants felt that contraception is unacceptable for adolescent girls, believing they are a tool for birth spacing, but are harmful to health and future childbearing in nulliparous girls.

*“I say that contraceptives are dangerous for someone who has not given birth before because in future they may not be able to bear children. I tell them it’s good to start using contraception when they are married and have young ones already.” M/FG; YCA01.*

Girl participants also reported receiving these messages from their parents and others in the community very clearly.

*“I was taught that if you start using contraception methods when you are too young you damage the uterus.” 14-year-old girl; XGC02.*

The majority of M/FGs and initiation counselors reported that they do not discuss contraception, including condoms, with adolescent girls because they feel it is harmful, inappropriate and/or will encourage sex. Among initiation counselors, the majority (9/10) report that they either do not teach or explicitly discourage birth control methods in initiation ceremonies because they lack knowledge and/or do not think it is appropriate.

*“Telling a girl aged 10-18 about contraception and condom use is like [saying] you are free to do it [have sex]… she will know that she is safe and will be more sexually active, and will become a prostitute.”* M/FG; YAA01.

A majority of girl participants viewed contraceptives and/or condoms as acceptable; and among those, access and provider attitude were reported as problems.

*“Doctors they do shout at them [girls seeking contraceptives] since they are young.”* 15-year-old girl; XKA03.

Overall, family planning is a topic about which girls reported both lacking in information and wanting more.

*“Condom use and contraceptive methods? I do not know anything and have not been taught, but it is necessary to teach girls how to prevent pregnancy.” 13-year-old girl; XBB01.*

### SRH education starts too late

SRH teaching often starts after girls have become sexually active, which some girls and M/FGs reported occurs as early as age eight or nine. Many M/FGs reported providing SRH information to their girls after age 15 and as late as 18. The girls themselves reported wanting information before sexual debut in order to make better decisions.

*“I never learned about these things until in hospital after giving birth, which is too late.”* 18-year-old girl; XKC02.

The most common reason cited by M/FGs for delaying provision of SRH information is their concern that it will promote sexual activity. As a result, they reported waiting until “changes” in the girls signaling sexual debut are observed, such as coming home late or a having a “rude” attitude, to initiate discussions about sex.

*“We just notice the change in behavior, coming late at night at home, stubbornness, and we know she has started sleeping with men. I call her and advise her.” M/FG; YCA02.*

### Initiation practices that encourage sexual activity

Roughly half of the M/FGs and girls interviewed reported a negative view of initiation ceremonies and initiation counselors as sources of information, primarily because some still advise “cleansing,” which is to practice sex. Three of the 10 initiation counselors reported that they still teach girls how to satisfy a man sexually and encourage practice after the ceremony. Although the majority of counselors claimed they do not encourage girls to practice sex, they reported knowing others who do.

*“When that girl comes out of the initiation camp she will begin having sex because that is what she has been taught. They are taught how men perform in bed.”* Initiation counselor; ZDB01.

Most religious initiation counselors also reported that they no longer encourage sexual cleansing and indicated that they do not use anatomical terms or explain specific acts because it is “pornographic” or not appropriate. This differed from what was reported by parents and girls, who suggest that many counselors are still encouraging sexual cleansing.

*“The church doesn’t allow that [sexual education]. But the [religious] counselor, just because they come from the villages, they normally fix in a little tradition… we know that normally whenever they say initiation ceremony, normally it’s something to do with ‘bed work.’”* M/FG; YWA02.

## Discussion

Taken together, the results of this study reveal conditions that perpetuate high rates of adolescent pregnancy in the region, including beliefs that contraception is unacceptable for nulliparous girls, and gender norms around the exchange of money and gifts for sexual intercourse that is often unprotected, also a risk factor for HIV and other sexually transmitted infections (STI). However, our study findings also offer insights on opportunities for early pregnancy and STI/HIV prevention that are applicable in both the study setting, as well as other similar settings with high rates of poverty and gender inequality.

Participants almost universally agreed that childbearing before age 18 could be harmful to the health of mother and baby, that female educational attainment is important for a girl’s future, and that early pregnancy usually marks the end of a girl’s schooling. Yet, interviewees also reported that transactional sex and childbearing before age 18 is very common in the village*.* Such societal contradictions are typical of social change processes and can be especially salient in the realm of sexuality [23–25]. IEC strategies that acknowledge and skillfully navigate these contradictions have the strongest potential for mobilizing the range of actors influential in girls’ lives and creating an enabling environment to promote their health [26–28].

As has been reported elsewhere in sub-Saharan Africa, our study shows poverty and gender norms provide the underlying context for adolescent unprotected sex, transactional sex, and resulting high rates of pregnancy and HIV infection [29, 30]. While adolescent girls do exchange sex for money and gifts, ethnographic approaches to understanding this exchange highlight its variegated nature as being far more complex than a simple market transaction [31, 32]. Both sex and money are considered important expressions of love. Providing for women with whom one is having a sexual relationship is an important marker of masculinity, and women receiving support from a sexual partner is a normative expectation and a sign of being valued [31, 32]. As a result, IEC programming will be more successful when situated within a socio-ecological framework, taking these contextual factors into account so that girls can successfully act on acquired SRH knowledge [29].

Notably, our study identifies parents as gatekeepers to their girls’ access to SRH information and services. While evidence from sub-Saharan Africa regarding family decision-making dynamics is sparse, it appears that parent-child communication in the African setting on topics such as sexual relationships, family planning, and HIV/STI prevention, is limited. Barriers include lack of parental knowledge, reliance on schoolteachers, and a perception that talking about sexuality encourages sex. Yet evidence shows that such communication is associated with decreased levels of sexual risk taking among adolescents [33], and that with support to develop parental responsiveness, parents can communicate effectively on these topics [34]. This suggests that in rural Malawi and similar settings, parental engagement must be a cornerstone of any adolescent SRH IEC effort. Messaging for parents, and other adults in the community, could emphasize health and wellbeing as well as the “cost” of not providing contraceptives and condoms in terms of the economic impact of HIV/AIDS, birthing, childrearing, and missed years of schooling.

Consistent with evidence from other low-resource settings with low levels of school attainment, our study shows that contraception, and even condom use, is both discouraged and inaccessible to adolescent girls [35]. The fact that many M/FGs and initiation counsellors disapprove of condom use by adolescent girls, is particularly concerning. Girls themselves are more open to condoms and other forms of contraception, but report difficulties accessing these commodities, and encountering health workers who disapprove of contraception for adolescents. In addition to efforts to generate support among adults who are influential in girls’ lives, health services need to be accessible and adolescent friendly [36].

Malawi’s most recent National Sexual and Reproductive Health and Rights (SRHR) Policy (2017–2022) commits to repositioning family planning for all, including adolescents, as a crucial development strategy. Specifically, the policy recognizes that family planning impacts individual health, family financial wellbeing, and economic development at a community and national level. Key to achieving this goal, both Malawi’s SRHR policy and its National Youth Friendly Health Services Strategy (2015–2020) encourage the harmonization of health, education and youth ministry policies with the aim of improving youth awareness of and access to SRH services. For example, there is an apparent conflict between the Ministry of Health advocating for SRH services for youth, and the MoEST prohibiting distribution of commodities such as condoms within certain distance of a school [37]. The recognition and emphasis by the Government of Malawi to catalyze cross-sectoral coordination is very encouraging and important. Furthermore, while a supportive policy environment is necessary, it is insufficient to ensure translation to implementation of programs and services on the ground. Inadequate facilities, stockouts of medicines and equipment, and healthcare worker shortages—particularly those trained in adolescent-friendly care—are impediments to delivery of YFHS, especially in rural areas.

Initiation ceremonies encourage a norm of sexual debut at puberty. The results of this study indicate that initiation counselors often identify as both church-affiliated and traditional, and that both types of counselors teach sexual cleansing and sexual satisfaction of male partners, making the distinction between the two unclear. The situation is paradoxically compounded because counselors do not educate girls on sexual health because they feel it is obscene or inappropriate. The ceremonies are an important social custom and are valued as a means of teaching traditional song and dance and transmitting cultural values such as respect for elders, and care and loyalty to the community. As such, they have the capacity to become an important platform for accurate and relevant SRH information [19]. Transforming these ceremonies has the potential to align stakeholders around common, correct messages and improve adolescent girls’ ability to avoid potentially risky sexual encounters. Given that only one of the initiators in this study had received formal training, a curriculum for counselors, both traditional and religious, is urgently needed. With the support of village chiefs, religious leaders, parents and other key stakeholders, initiation counselors could be trained and incentivized to present accurate and adolescent-friendly SRH information in an intervention that is closely monitored and evaluated.

Importantly, our results also show that SRH information for adolescents starts too late. Many girls aged 11 and younger in rural Malawi have started having sex, with most reporting becoming active at menarche (Bvumbwe A: Girls EMpowerment Program, Needs Assessment Report, 2016, unpublished). Reaching girls in early adolescence (10–14 years) – prior to sexual debut, before gender norms become entrenched, and while most girls are still in school – can be particularly effective [38–40]. The literature supports the notion that withholding information increases the risk of unwanted pregnancy [41]. And while initiation ceremonies may provide an opportunity to reach many girls at puberty, it remains a one-time, albeit culturally embedded, touch point that can supplement but not replace CSE, which is a sequential year, age-appropriate rights-based and gender-focused curriculum. The critical gap in CSE delivery to young adolescents has been documented elsewhere, including a UNESCO review of sexuality curricula in ten African countries, including Malawi [12]. Sexuality education that comes too late is culpable in perpetuating high rates of unintended pregnancy among adolescents. Policies supporting age-appropriate CSE beginning at least by standard 5 are essential.

While pregnancy in much of sub-Saharan Africa equates with the termination of a girls’ education, “Back to School” policies allowing return to school after pregnancy represent a sign of progress. An evaluation of these policies in sub-Saharan Africa including Malawi, however, identified barriers, such as lack of implementation, limited family support, as well as stigma and discrimination of pregnant girls, including those who have returned after childbearing, by administrators, teachers and peers at school. As a result, few girls in the setting actually return to school after pregnancy [12]. Increasing parental, teacher and community support for school enrollment retention policies, and/or considering a policy change to allow girls to remain in school during pregnancy, will be crucial to their success. Likewise, while eliminating secondary school fees is a positive step, given MoEST budget constraints and the need to maintain a degree of quality, it remains to be seen how this policy will be implemented, including what “administrative” fees may still apply and represent a barrier to students.

Out-of-school approaches can help reach adolescents who are not enrolled in school or can augment in-class CSE. For example, girl groups led by “outside” experts and role models who provide information and encourage questions were frequently described by girls, and M/FGs, in this study as a preferred source for SRH information. Many girls also reported that, while they understand education is important, they or their peers often fail to see a future other than motherhood and lose motivation to stay in school. The SRH and social learning literature suggests that facilitation by credible leaders and role models can improve SRH outcomes by developing girls’ aspirations for school completion and possibilities for income generation [34, 36, 42]. Girl group curricula presented in the context of life-skills, empowerment, and values-based learning, has been associated with reduced childbearing among adolescents, [43] as well as lower rates of HIV infection [41]. Incorporating an income-generation component (e.g., sewing, milling) could support scalability and sustainability of groups, increase girls’ empowerment, and reduce reliance on transactional sex [44, 45].

Finally, framing SRH IEC in terms of girls’ rights – to defend their own destiny and dignity with opportunities equal to those of boys -- is important for adolescent girls, and boys, no matter the intervention or the delivery platform. SRH IEC programs that go beyond conventional teaching to include gender norms and rights- and empowerment-based approaches – allowing girls to see themselves as equals in relationships, able to protect their own health, and capable of engaging actively in society – have been shown to increase girls’ status and agency in the community, as well as have a greater likelihood of reducing rates of STIs and unintended pregnancies [43].

Our formative research has several limitations. The research team relied on the village chiefs to provide lists of households that met criteria. Although researchers were able to choose households from lists provided, it is possible that the chiefs provided, even inadvertently, a selection of households biased against harmful practices and toward recognized healthy practices, e.g., girls who have remained in school and avoided pregnancy. Also, because we were unable to identify girls ages 10–12 for participation and because the scope of our work was limited to female perspectives, the results lack the experience of very young adolescents and males, important groups to target in order to influence gender equity and the SRH of girls.

## Conclusions

Our findings provide actionable information for policy makers and program developers and implementers. However, information specific to early adolescents, male voices, and family decision-making and communication dynamics is needed. While the Global Early Adolescent Study [38] is shedding new light on young adolescents, gender norms and how those impact sexual risk, behaviors and outcomes, further formative research is needed to address the remaining gaps in understanding how to most effectively reach early adolescents with IEC interventions to promote positive norms and SRH. Additionally, investigation to understand male perceptions is needed to guide messaging and delivery of male-targeted IEC programs in support of adolescent SRH generally and to reduce early and unintended pregnancy specifically. Finally, because parents are potential sources of SRH information themselves, as well as critical gatekeepers to other sources of IEC and services, a better understanding of communication and decision-making within the family context would help elucidate this important aspect.

Our findings have several implications. First, inequitable gender norms, late provision of SRH information, and misinformation about contraception and sexual practices, including during initiation ceremonies, appear to be important drivers of unprotected sexual intercourse among adolescent girls in Mulanje. These factors are amenable to SRH IEC and community sensitization and mobilization interventions, especially when important stakeholders such as parents are engaged in a shared understanding of their daughters’ SRH and when girls are exposed to role models who can help them develop aspirations for a healthy future. Lack of access to adolescent-friendly SRH services appears to be a barrier to HIV/STI and unintended pregnancy prevention in this study setting. A health sector response is imperative, and non-governmental organizations can play important roles in filling gaps in adolescent-friendly service provision, especially in hard-to-reach areas. Poverty is a more distal, yet important, determinant of adolescent risky sexual practices, especially among girls. Though an entrenched issue requiring a multi-sector response, poverty and socioeconomic inequity alleviation will be essential to achieve long-lasting impacts on adolescent wellbeing. Efforts to improve access to, adolescent demand for, and community support of SRH education and services; debunk community-wide misinformation and rumors about contraceptives; leverage initiation ceremonies as opportunities for accurate information and void of harmful practices; engage parents and other stakeholders to create an enabling environment; and provide timely, accurate SRH IEC in a rights-based framework that also seeks to develop girls’ aspirations for a healthy future can play important roles in reducing early and unintended pregnancy and STIs and contributing to breaking the cycle of poverty.

## Abbreviations

CSE: Comprehensive sexual education
GAIA: Global AIDS Interfaith Alliance
HIV: Human immunodeficiency virus
HIV/AIDS: Human immunodeficiency virus/acquired immunodeficiency syndrome
IEC: Information, education and communication
M/FG: Mother/female guardian
MoEST: Ministry of Education, Science and Technology
NGO: Non-governmental organization
SRH: Sexual and reproductive health
STI: Sexually transmitted infection
UNESCO: United Nations Education, Scientific and Cultural Organization
